# Predictable Peptide Conjugation Ratios by Activation of Proteins with Succinimidyl Iodoacetate (SIA)

**DOI:** 10.3390/mps1010002

**Published:** 2017-09-25

**Authors:** Ioana M. Abbas, Timm Schwaar, Frank Bienwald, Michael G. Weller

**Affiliations:** 1 Federal Institute for Materials Research and Testing (BAM), Division 1.5 Protein Analysis, Richard-Willstätter-Strasse 11, 12489 Berlin, Germany; ioana.abbas@gmail.com (I.M.A.); timm.schwaar@gmx.de (T.S.); bienwalf@hu-berlin.de (F.B.); 2 Humboldt-Universität zu Berlin, School of Analytical Sciences Adlershof, Unter den Linden 6, 10099 Berlin, Germany; 3 Humboldt-Universität zu Berlin, Department of Chemistry, Brook-Taylor-Str. 2, 12489 Berlin, Germany

**Keywords:** bioconjugate, carrier protein, immunogen, hapten, linker, antibody drug conjugate (ADC), drug-to-antibody ratio (DAR), conjugation ratio, conjugation density, carrier load, click reaction

## Abstract

The small heterobifunctional linker succinimidyl iodoacetate (SIA) was examined for the preparation of peptide–protein bioconjugates with predicable conjugation ratios. For many conjugation protocols, the protein is either treated with a reductant to cleave disulfide bonds or is reacted with thiolation chemicals, such as Traut’s reagent. Both approaches are difficult to control, need individual optimization and often lead to unsatisfactory results. In another popular approach, a heterobifunctional linker with a *N*-hydroxysuccinimide (NHS) and a maleimide functionality is applied to the protein. After the activation of some lysine ε-amino groups with the NHS ester functionality, a cysteine-containing peptide is attached to the activated carrier protein via maleimide. Particularly, the maleimide reaction leads to some unwanted byproducts or even cleavage of the linker. Many protocols end up with conjugates with unpredictable and irreproducible conjugation ratios. In addition, the maleimide-thiol addition product should be assumed immunogenic in vivo. To avoid these and other disadvantages of the maleimide approach, we examined the known linker succinimidyl iodoacetate (SIA) in more detail and developed two protocols, which lead to peptide–protein conjugates with predefined average conjugation ratios. This holds potential to eliminate tedious and expensive optimization steps for the synthesis of a bioconjugate of optimal composition.

## 1. Introduction

The attachment of peptides to proteins and other biopolymers is one of the most important methods for the synthesis of bioconjugates. Many textbooks and excellent reviews are available in this field [1,2].

Peptide–protein conjugates are used for immunization purposes, immunoassay development and more recently for the preparation of antibody drug conjugates (ADCs) [3,4,5,6]. Unfortunately, the yield of such conjugation reactions is variable and hence, tight quality control of such products is necessary. The analytical characterization of such conjugates is demanding and in most cases only empirical tests or semi-quantitative assays are used. The first property to be tested is the conjugation density, also known as conjugation number, conjugation ratio, haptenic density, hapten density, degree of modification, payload occupancy, or drug-to-antibody ratio (DAR) [7]. Quite a few methods have been developed to determine this parameter, such as radioactive labelling, amino acid determination (e.g., introduction of norleucine), elemental analysis, electrophoresis and others. However, today, the use of matrix-assisted laser desorption/ionization time-of-flight mass spectrometry (MALDI-TOF-MS) seems to be the most convenient and precise method to determine the conjugation density of peptide-carrier and other conjugates [8,9]. High-resolution mass-spectrometry with electrospray ionization (ESI) is another powerful method. Although the resolution of different conjugation levels is possible in some cases, it is rarely performed due to expensive instrumentation and complex data evaluation. Nevertheless, this is one of the rare methods that offer information about the conjugation distribution, which usually is quite broad, even under controlled synthesis conditions. Furthermore, hydrophobic interaction chromatography (HIC) is used for the determination of DAR [10,11], particularly in the ADC field. The heterogeneity of the products is a major issue of such bioconjugates. Even if the number of peptides per carrier protein would be the same, the sites, where the peptides would be attached, may be variable. This is the case for nearly all methods of chemical conjugation. Site-specific conjugation would be highly desirable and some approaches have been developed, already. Unfortunately, either a recombinant protein with site-specific mutations is necessary or other quite complex approaches would have to be applied. Therefore, even in the field of pharmaceutical products, e.g., for PEGylated proteins or antibody drug conjugates (ADCs), statistically attached conjugates are used frequently. The site of attachment is difficult to determine in heterogeneous mixtures, since the chromatographic or electrophoretic separation is very difficult, if not impossible in medium- to high-density conjugates, for which a very high number of isomers and congeners are formed. With digestion methods, mean conjugation site occupancies can be determined by liquid chromatography coupled to tandem mass spectrometry (LC-MS/MS), such as the examination of posttranslational modifications (PTMs). Recently, quite a few methods have been developed using some variants of “click chemistry” to achieve a site-specific conjugation. However, in most cases, a non-natural peptide side-chain has to be introduced by some other means and hence, the problem is only shifted. Another issue of many click-chemical protocols is the poor water-solubility of some reagents and the introduction of large, hydrophobic groups, rendering the protein highly immunogenic for the linker and highly prone to precipitation and denaturation. However, the haloacetyl-thiol reaction based on succinimidyl iodoacetate (SIA) and related reagents, also known as thiol-halogen ligation, can definitely be regarded as a click reaction [12].

Surprisingly, there is only scarce information about the optimal conjugation density for a specific application [13,14,15,16]. Several points should be considered. First, the solubility of the carrier protein and its conjugate is important. Therefore, bovine serum albumin (BSA) is a very popular carrier protein. Precipitation occurs only at quite high conjugation densities. In addition, the use of organic solvent mixtures to improve the solubility of the reagents is better tolerated by BSA than by other proteins. For immunogens, there seems to be an optimal conjugation density of about one-third to one-half of the lysines available, which translates to about 10–15 peptides per BSA molecule. Lower levels might simply make the peptide epitope too rare to be sufficiently immunogenic and the carrier epitopes would be dominant. Higher levels might lead to precipitation [17] and in addition, might lead to antibodies recognizing more than one peptide in one binding site. This might lead to “non-competing” antibodies, being essentially useless for most applications. To produce protein conjugates for immunoassays, a low conjugation density is desirable in many cases. The main reason might be the bivalent structure of immunoglobulin G (IgG), which prefers multivalent complex formation where possible. In this case, the competition with a monovalent analyte is highly unfavorable. This seems to be an inherent advantage of peroxidase-based conjugates, which display, due to the low number of accessible lysines, a functionally monovalent behavior. Except of this special case, it would be very convenient if the stoichiometrically calculated reagent amount would directly define the intended conjugation density. This is only possible, if the conjugation chemistry is reproducible and of a high yield, which is not the case with most chemistries in use. Often, a high excess of small-molecule reagent is used, which makes the production of conjugates of low or medium density a matter of chance.

**Figure 1:**
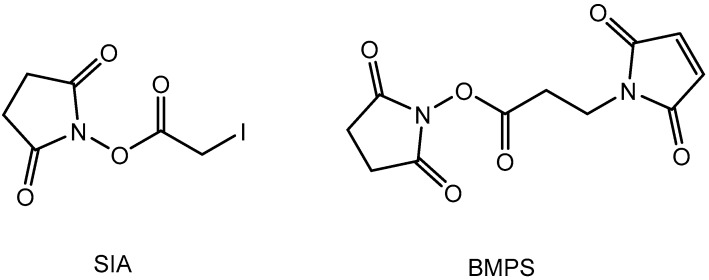
Structures of the activation reagents succinimidyl iodoacetate (SIA) and *N*-(β-maleimidopropyloxy) succinimide ester (BMPS).

The conjugation of peptides to proteins can be achieved by several chemistries. A very traditional approach is the activation of the protein by glutaraldehyde, and the subsequent addition of a peptide with at least one free amino group, including the N-terminus. The formed imines may be reduced by borohydride reagents to stabilize them and to prevent any reverse reactions. These bioconjugations, which can be carried out in one-pot or two-step protocols, are quite reliable and hence are used frequently. However, the structure of the products is heterogeneous. To achieve more defined conjugates, heterobifunctional linkers have been introduced. Many of them contain a *N*-hydroxysuccinimide ester function (NHS) and a maleimide function (Figure 1). The linker in between can vary in length and structure. The NHS functionality leads to the attachment of the linker to some lysines at the carrier protein or enzyme [18]. The free maleimide function can be attacked by a thiol(ate), mostly delivered by a cysteine. This amino acid is often added to the desired peptide sequence, solely to enable this kind of conjugation. Only in rare cases, the used peptide already possesses a suitable cysteine in the sequence, which can be used for conjugation. Sometimes, the thiol is generated by reduction/cleavage of a disulfide bridge in a peptide or protein, which however, can lead to severe and often unwanted changes in secondary and tertiary structure. Unfortunately, maleimide conjugations suffer from some significant disadvantages. They tend to generate some side reactions, such as hydrolysis. These reactions can lower the conjugation yield significantly and may lead to unstable conjugates [19]. Furthermore, solvent-accessible maleimide-thiol adducts show a fast loss of the linker via maleimide exchange with reactive thiols in blood plasma [20,21]. Recently, some advanced maleimide (“next generation maleimide”) linkers have been presented in the literature [22,23], which showed improved stability of the respective conjugates. However, these linkers seem to be not available commercially.

In addition, it could be shown that the formation of diastereomers [24,25] leads to problems in structure analysis based on enzymatic digests and LC-MS. These isomers may lead to the formation of two chromatographic peaks for each affected peptide [26]. Furthermore, maleimide conjugates contain a quite rigid cyclic structure, which may be immunogenic, if used as an immunoconjugate. This is unwanted in most cases, since the maleimide is not part of the targeted epitope. In an interesting study [27], it could be shown that the widely used linker succinimidyl-4-(maleimidomethyl)-cyclohexane-1-carboxylate (SMCC) proved to be highly immunogenic and even suppressed an antibody response against a carbohydrate antigen. A more detailed examination showed that anti-linker antibodies recognized terminal hydrolyzed maleimides, as well as internal ones. In comparison, a bromoacetamido linker, very similar to the SIA linker examined in this paper, elicited a significantly lower anti-linker and a higher anti-hapten response. In a later paper [28], it was also stressed that thiosuccinimides formed from maleimides are immunogenic themselves. Alkylthioethers, which are also the product of SIA conjugations, have been recommended as “nonimmunogenic linkers”. Finally, many hydrophobic maleimide linkers easily lead to the precipitation of the respective activated protein [29], which limits the number of haptens, which can be attached. The use of more hydrophilic sulfosuccinimide derivatives do not help much to resolve this issue, since the sulfo-group is lost during the conjugation.

Haloacetates have been used as derivatization reagents in protein chemistry for quite a while. The first reports of the reaction of sulfhydryl groups with iodoacetic acid were published in 1933 [30] and 1934 [31]. Probably the most frequently used reagent today is iodoacetamide, which is used for the stabilization of thiol groups in proteomic workflows based on mass spectrometry [32]. In addition, quantitative protein analysis based on isotope-coded affinity tags (ICAT) [33], metal-coded affinity tags (MeCAT) [34] and other related methods are highly dependent on the efficient alkylation of thiols. Thousands of papers describing this approach can be found in the literature. Other applications are labeling reactions with iodoacetyl-groups attached to the respective label, such as fluorescent dyes, metal containing labels, mass tags and others. In this case, some free thiols must be (made) available at the protein or peptide. This can be achieved, e.g., by partial reduction/cleavage of disulfide bonds or thiolation of amines by Traut’s Reagent (2-iminothiolan), succinimidyl acetylthioacetate (SATA), succinimidyl 3-(2-pyridyldithio)propionate (SPDP), *S*-acetylmercapto-succinic anhydride, and others.

The opposite strategy, the attachment of haloacetates to a protein to achieve a thiol-reactive site, is rare in the literature. This is even more surprising if it is considered that the majority of peptide conjugations today are performed via cysteine derivatives, which are easily and economically available by solid-phase peptide synthesis (SPPS).

In an early work, Rector et al. used succinimidyl iodoacetate for the preparation of IgG–ovalbumin conjugates [35]. Later, Inman proposed the synthesis of haloacetyl reagents in a short paper [36]. He already mentioned some drawbacks of maleimides used for thiol derivatization, such as hydrolytic degradation. In addition, some advantages of haloacetyls were discussed, such as much slower hydrolysis and weak immunogenicity, which avoids the formation of antibodies against the bridging structure [37].

A few papers proposing succinimidyl iodoacetate (SIA, Figure 1) as a general conjugation reagent were published by the group of Houen [38,39,40,41,42]. Carboxymethyl-cysteine after acidic hydrolysis was used for the determination of the conjugation density with cysteine-containing peptides and it could be proven that the respective conjugates were suitable for immunization purposes.

In contrast to the maleimide approach, which gained widespread popularity, e.g., for ADCs [43,44], succinimidyl iodoacetate (SIA) conjugation was rarely used in practice. Motivated by the documentation of maleimide instability, product degradation, unwanted immunogenicity [17,37,45,46,47] and isomer formation of these linkers [19,20,48], we decided to reexamine succinimidyl iodoacetate (SIA) as an alternative reagent in more detail. It could be shown that this bioconjugation approach is highly reproducible, can lead to conjugates of predefined average conjugation density and might be superior to the standard maleimide chemistry in many cases.

## 2. Materials and Methods

### 2.1. Chemicals and Reagents

All solvents (methanol X948.2, acetonitrile HN44.2) used were high performance liquid chromatography (HPLC) grade, purchased from Carl Roth (Karlsruhe, Germany). Distilled water was obtained from a Milli-Q water-purification system (Millipore, Bedford, MA, USA). The peptide DTHFPIC was obtained from peptides & elephants (Henningsdorf, Germany). The linkers *N*-(β-maleimidopropyloxy) succinimide ester (BMPS, 22298) and succinimidyl iodoacetate (SIA, 22349) were purchased from Life Technologies (Darmstadt, Germany). Bovine serum albumin (BSA) (A2058), disodium hydrogen phosphate dihydrate (71643), sodium dihydrogen phosphate dihydrate (71505), sodium chloride (71376), citric acid monohydrate (33114) and trisodium citrate dihydrate (71402) were obtained from Sigma-Aldrich (Taufkirchen, Germany). α-Cyano-4-hydroxycinnamic acid (CHCA, 8201344) was purchased from Bruker-Daltonik (Bremen, Germany).

### 2.2. MALDI-MS Characterization

After the desalting procedure, the aqueous conjugate solutions were further diluted with lab water to obtain concentrations within the desired concentration range of 10 pmol/μL (0.01 mM) to 100 pmol/μL (0.1 mM). All matrix-assisted laser desorption/ionization time-of-flight (MALDI-TOF) mass spectra were acquired on a Bruker Reflex III MALDI mass spectrometer (Bruker-Daltonik, Bremen, Germany) operated with a nitrogen laser and at 20 kV acceleration voltage in linear positive mode. α-Cyano-4-hydroxycinnamic acid matrix was freshly prepared (10 g/L in water/ACN/TFA (33%/66%/0.1% *v*/*v*/*v*) and pre-coated with a droplet of 0.75 μL. An amount of 0.75 μL of protein sample was added to the same spot and dried for 1 h.

### 2.3. Solvolysis and Hydrolysis of SIA and BMPS

UV-visible absorbance measurements were performed on an Evolution 220 UV-Visible Spectrophotometer (Thermo-Fisher Scientific, Dreieich, Germany). A quartz cell (Hellma Analytics, Müllheim, Germany) with 10 mm light path was used. Solutions of the cross-linkers SIA and BMPS in methanol/acetonitrile were added to a buffer solution for a final concentration of 0.03 mg/mL. Phosphate buffer (12 mM sodium phosphate, 137 mM sodium chloride) was used for the pH range 6 to 9 and citrate buffer (35 mM citric acid, 65 mM trisodium citrate) for pH 5. Different pH values were achieved by adjusting the pH with NaOH 20% and HCl 20%.

### 2.4. Activation of Proteins

BSA was dissolved at a concentration of 2.5 mg/mL in PBS buffer (12 mM sodium phosphate, 137 mM sodium chloride, pH 7.4) in a glass vial. Different pH values were achieved by adjusting the pH with NaOH (20%) and HCl (20%). SIA was dissolved in acetonitrile at 6 mg/mL immediately before use. For each experiment, 1 mg of BSA was used. The cross-linker solution was added to the BSA solution under stirring in the specified molar ratio of BSA:SIA. The mixture was incubated for 30 min at room temperature. Prior to MALDI-MS analysis, the sample was desalted using Vivaspin 2 Hydrosart, 30 kDa (Sartorius, Göttingen, Germany) following the manufacturer’s instructions.

### 2.5. Conjugation of Peptides to Activated Proteins

The peptide DTHFPIC was dissolved in distilled water to give a 10 mg/mL stock solution. Aliquots of 100 µL were stored at −20 °C. Freshly thawed stock solution was used within a few days. The peptide solution was added to the activated BSA solution in the specified molar ratios of BSA:peptide and incubated overnight. The same desalting procedure described earlier in the activation of the protein was applied.

## 3. Results

### 3.1. Solubilization of SIA in Organic Solvents

**Figure 2:**
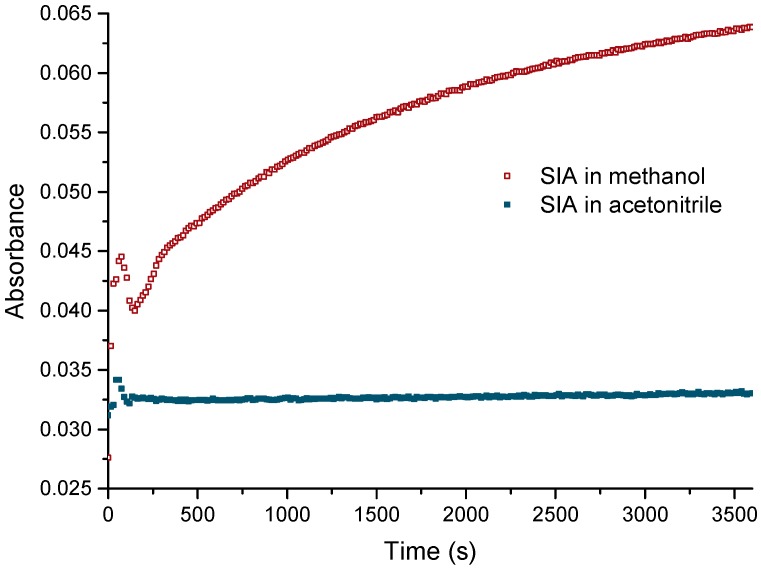
Solvolysis of succinimidyl iodoacetate (SIA) by methanol and acetonitrile. Monitoring of UV absorbance at 260 nm.

The activated ester succinimidyl iodoacetate (SIA) is a non-charged compound and shows a limited solubility in aqueous buffers. Therefore, the stock solution of SIA was prepared in organic solvents. Methanol would be preferable due to its excellent compatibility with proteins in the form of diluted, aqueous solutions [49,50]. Unfortunately, the reactivity of SIA is too high; as such, it degrades significantly even in dry methanol. This indicates a strong electron withdrawing effect of the halogen atom at the acetate group, which makes the NHS ester much more reactive than in usual NHS reagents. This should be considered when SIA is used as a derivatization reagent. Based on these results, acetonitrile was tested as a solvent for the stock solution. As expected, the dry, non-nucleophilic solvent did not show any significant reactivity towards SIA (Figure 2). The solvolysis of SIA was monitored by UV spectrometry at 260 nm [51], which is the absorbance maximum of free *N*-hydroxysuccinimide (NHS). This simple method is suitable for the monitoring of NHS in pure, UV non-absorbing samples, in contrast to other cases, where chromatographic methods are required [52]. From Figure 2, it is obvious that acetonitrile is a preferred solvent for SIA stock solutions.

### 3.2. Hydrolysis of the Ester at Different pH Values

Protein conjugations cannot be performed in pure organic solvents; therefore, aqueous buffers are needed for this purpose. However, SIA shows quick hydrolysis in water, which must be taken into consideration. To achieve a more quantitative examination of SIA hydrolysis, the half-life of SIA at different pH values were determined (Table 1a). The results show that at a pH of 7 or above, the active ester is completely hydrolyzed after 5 min (10 half-lifes). In contrast, N-(β-maleimidopropyloxy) succinimide ester (BMPS), as an example of an ordinary NHS ester reagent, is reactive for more than 1 h, even at a pH of 8 (Table 1b).

### 3.3. Activation of Proteins

For these experiments, bovine serum albumin (BSA) was chosen as a model protein. It is known that from its 59 lysines, only 30–35 are accessible for NHS ester derivatization [2]. In contrast to most conjugation studies, the activation step and the peptide attachment were examined separately.

**Figure 3:**
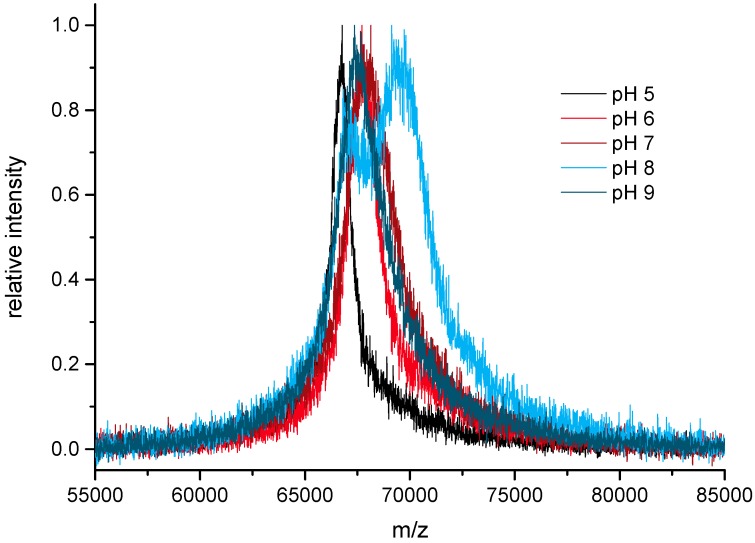
Influence of pH on activation level at constant SIA excess.

First, the optimal pH of this activation was explored. It could be shown (Figure 3) that the highest conjugation ratios were achieved at pH 8. The bimodal distribution is suspected to have been caused by inefficient mixing. Even at pH 6 and 7, good conjugation ratios were obtained. At pH 9, the conjugation was poor, probably due to nearly instantaneous hydrolysis. As a compromise, a pH of 7.4 was chosen for most of the further experiments to achieve optimal compatibility even with sensitive proteins.

**Figure 4:**
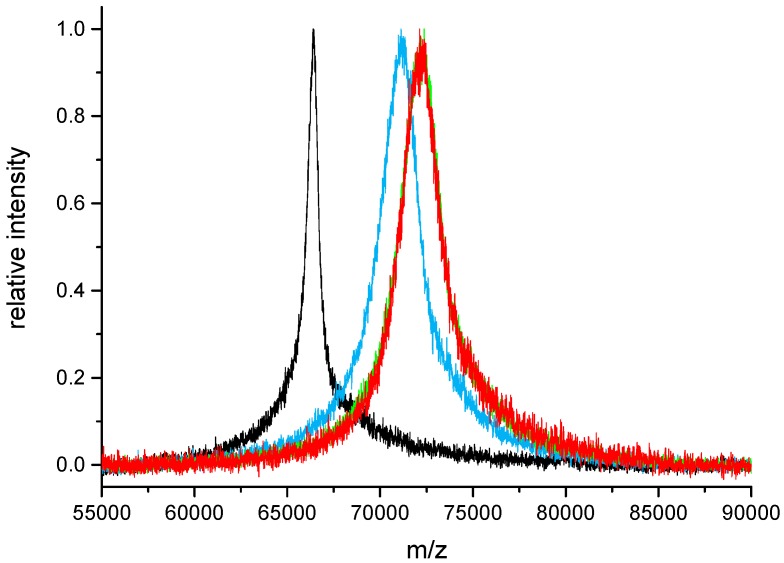
Saturating SIA activation of BSA in several steps (matrix-assisted laser desorption/ionization time-of-flight (MALDI-TOF) mass spectra). A 60-fold excess of SIA, pH 8.3, black: bovine serum albumin (BSA), blue: BSA + 2 steps, green: BSA + 3 steps, red: BSA + 4 steps of activation).

Due to the limited solubility of SIA in aqueous buffers and its quick hydrolysis, a complete derivatization of all available lysine ε-amino groups of BSA in one step was not achieved. For activation levels higher than 18 (pH 7.4, 60-fold excess of SIA relative to BSA), repetitive additions of SIA solution were performed. With several SIA additions (Table 2), a maximum conjugation level of 35 was achieved, which is in very good accordance with literature values of 30–35 [2]. In Figure 4, conjugation steps 0, 2, 3 and 4 are depicted. It can be shown that at a pH of 8.3, three steps of activation are needed for essentially saturating activation. It should be stressed that for most applications (BSA conjugation rates below 15), a single activation step is sufficient, even in the Protocol I “Excess of SIA”.

In Figure 4, it is shown that three activation steps of SIA addition are sufficient to achieve an essentially complete saturation of all possible conjugation sites (>30 mol/mol). In Table 2, a complete series of experimental activation levels is listed. In a single step, about 18 mol/mol are attained, which should be sufficient for the vast majority of all applications.

In other experiments, a different excess of SIA (20×, 40×, 60×) relative to BSA was used for activation. Activation levels of 8–19 were achieved in a single step (Table 3).

### 3.4. Peptide Conjugation, Excess of SIA (Protocol I)

Two-step conjugations can be performed under different regimes. With the exception of the conceptually trivial 1:1 (linker:peptide) approach, which is quite demanding in practice, and the other trivial option, excess linker and excess peptide, which most often leads to insoluble precipitates, there are two particularly useful variants:

Protocol I: Excess of SIA, with peptide-limited conditions.

Protocol II: Excess of peptide, with linker-limited conditions.

**Figure 5:**
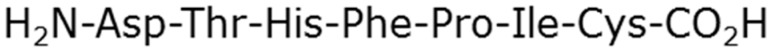
Sequence of the model peptide (Molecular Weight (MW) = 831.94 g/mol) derived from seven amino acids of the N-terminus of hepcidin-25.

**Figure 6:**
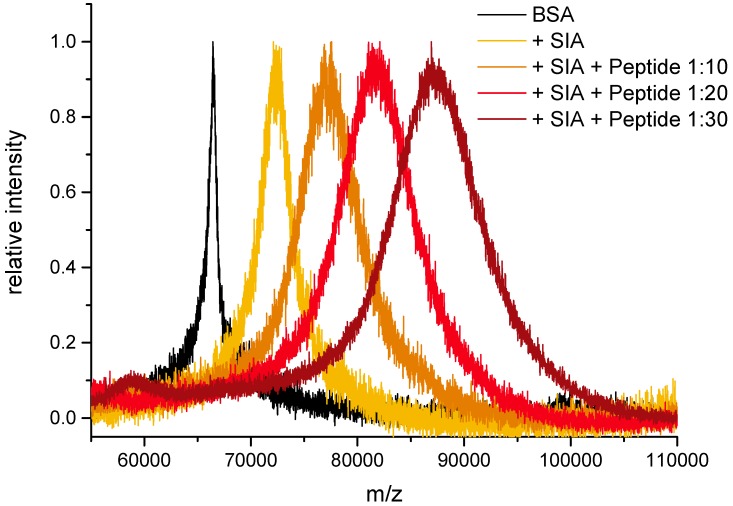
Protocol I (peptide limited): Conjugation of cysteine-containing model peptide with SIA-activated serum albumin (BSA). The + SIA spectrum shows BSA with a three-fold activation step (saturation). The ratio refers to BSA:peptide.

In the experiments shown here, the N-terminus of hepcidin-25 containing a C-terminal cysteine was used as a model peptide (sequence in Figure 5). For this purpose, BSA was activated to a high (saturation) level by repeated reaction with SIA. Subsequently, the activated carrier protein was conjugated with different ratios of cysteine-containing peptide. It could be shown that the conjugation yield was proportional, which makes the synthesis of conjugates with predefined average conjugation ratio quite straightforward (Figure 6 and Table 4).

In Table 4, it can be seen that an essentially quantitative and hence predictive conjugation of the peptide is achieved. It has to be considered that some activated sites are left over, which leads to a high level of lysine derivatization. This might be relevant for tryptic digests and mass spectrometric studies. An inactivation step may not be necessary in most cases. Otherwise, thiols, such as cysteine, cysteamine, thioglycerol or glutathione (reduced), might be applied. In the long term, the remaining iodide groups should slowly hydrolyze to hydroxy groups or might lead to intramolecular cross-links.

### 3.5. Peptide Conjugation, Excess of Peptide (Protocol II)

**Figure 7:**
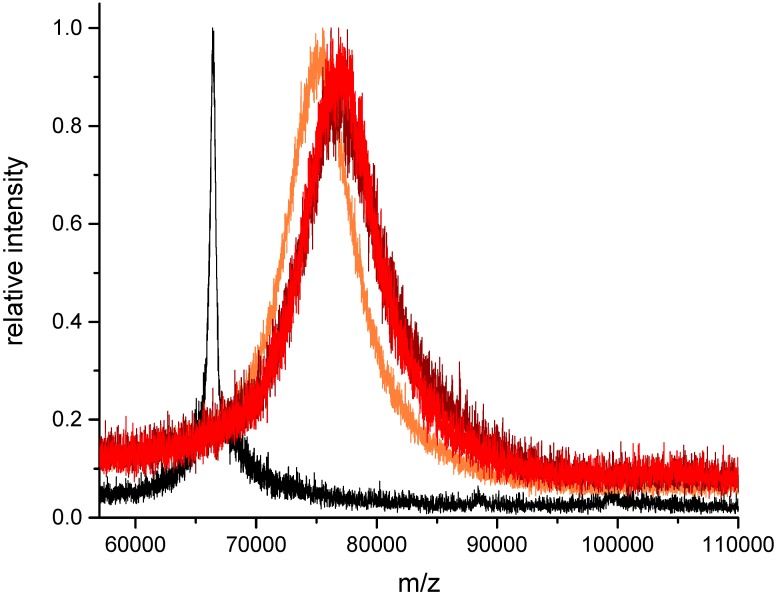
Protocol II (activation limited): Conjugation of cysteine-containing model peptide with SIA-activated serum albumin (BSA). BSA: black, BSA + SIA + Peptide 1:20: orange, BSA + SIA + Peptide 1:30: brown, BSA + SIA + Peptide 1:40: red.

In the case of less pure cysteine-peptides with an unknown fraction of disulfide dimers, the application of the second protocol should be considered. Here, the level of SIA activation is limiting and determining the conjugation level. Any optimization is performed with the cheaper activation reagent. For the subsequent reaction with the cysteine-containing peptide, a moderate excess of peptide (e.g., 2× relative to activated sites) should be applied. In Figure 7, an example is shown. BSA has been activated with a nominal SIA excess of 1:40 to achieve an activation level of 10.8. This was verified by MALDI-TOF-MS. In the second step, a peptide excess of 1:20, 1:30 and 1:40 was tested, which corresponds to an excess of 1:2, 1:3 and 1:4 relative to the activated sites. It is obvious that in contrast to Figure 5, where the increase of the peptide amount leads to a proportionally higher conjugation level, Protocol II results in a maximum level, which is defined by the SIA activation step. The 1:20 amount leads nearly to the calculated level (10.0), whereas the peaks of the 1:30 and 1:40 amount are slightly shifted to higher masses (12.5 and 12.4). The reason might be either an inaccuracy in the determination of the SIA activation level or the indication of some non-selective reactions, e.g., by thiol exchange. However, for most experiments, this small difference should be of no relevance.

## 4. Discussion

SIA has many advantages for peptide conjugations. Disulfide-cleavage or thiolation of the protein is not required. These steps are poorly controllable and often lead to crosslinking, oligomerization, aggregation and precipitation. In contrast, the “thiolation” of peptides is straightforward by introduction of cysteine during automated solid-phase peptide synthesis (SPPS). Subsequent HPLC removes nearly all impurities. In addition, the formation of peptide dimers is much less critical than the formation of protein oligomers. Peptide dimers may reduce the conjugation rate in Protocol I, but not in Protocol II. Protein oligomers, which may be formed after traditional thiolation steps, often contaminate the final product and are difficult to remove quantitatively, or if they precipitate, the product yield is impaired. In contrast, peptide dimers and other small byproducts will be removed by a desalting or buffer exchange step, which is applied at the end of the conjugation anyway. 

SIA is a cheap reagent and readily available. The reactivity of its NHS ester moiety is very high and hence the activation of the protein is quite fast and can be performed even at neutral pH. Some light sensitivity of SIA was reported.

The structure of SIA is also quite advantageous. The linker is so small that it is essentially “invisible” to the immune system. These “stealth” properties lead to a very low immunogenicity and hence, the production of unwanted linker antibodies can be minimized. In some papers, it could be shown that other popular linkers and activation reagents, such as carbodiimides, lead to very strong immune responses against the respective reagent or its byproducts, which can cause unexpected problems, e.g., in screening protocols or immunoassay development. Furthermore, the SIA linker does not lead to additional diastereomers, which are the reason for unwanted double peaks in chromatographic peptide separation, e.g., in proteomics protocols. The product of the thiol alkylation is very stable and is not prone to hydrolysis, rearrangement, reverse reaction or enzymatic degradation. Only strong oxidants should be avoided. According to several studies, maleimides, which are preferentially used for cysteine-based conjugations today, are much more sensitive to the formation of unwanted byproducts or cleavage.

In this paper, it could be shown that peptide–protein conjugates can be synthesized with simple protocols based on the very small linker SIA, leading to clean products with a predictable conjugation density. However, this conjugation approach is not site-specific, if several reactive amino groups are available. In addition, the peak broadening in Figure 6 and Figure 7 shows that these reactions lead to a distribution of different conjugation densities (or DAR). Nevertheless, for most applications today, a conjugate with a defined and confirmed average conjugation density is fit for purpose. In addition, it should be considered that many site-specific approaches have other drawbacks, such as high cost, low yield, complicated production and the introduction of bulky hydrophobic groups, which reduce solubility and increase the tendency of aggregation. Furthermore, most of these site-specific constructs are highly immunogenic, which may severely limit their application in vivo.

Sometimes, a longer linker might be desirable. In this case, succinimidyl (4-iodoacetyl) aminobenzoate (SIAB) or sulfosuccinimidyl (4-iodoacetyl) aminobenzoate (Sulfo-SIAB) could be used. However, steric problems might be resolved in a cheaper and more reproducible way by standard modifications during the peptide synthesis, e.g., by attachment of one or several glycine residues. Most researchers are not aware of the problems with long, heterobifunctional linkers, which often show low conjugation yields and different conjugation kinetics depending on their structure. With the use of SIA, the conjugation steps can be expected to be fast and highly reliable.

For the conjugation of peptides to carrier proteins, two different protocols were presented. Protocol I uses a highly-activated protein to produce conjugates of different, but defined average conjugation densities. However, in this case, pure, and dimer-free cysteine-peptides should be used, to achieve the nominal conjugation level. The second protocol II is based on a carrier protein with limited SIA activation, which leads to a single predefined average conjugation density, irrespective of the dimer content or otherwise deviating concentration of the cysteine-peptide, if a sufficient excess of peptide can be assured. In the long term, such pre-activated carrier-proteins with a defined activation level might be offered commercially. Both approaches have their pros and cons, which are summarized below:

**Protocol I: Excess of SIA**
+ SIA conjugates are cheap and quick to prepare.
+ A single SIA conjugate can be used for peptide conjugates of different, controlled conjugation ratios.
+ Only low amounts of peptide are needed.
− Peptide should be free of cysteine dimers and peptide concentration needs to be known.
− Tris(2-carboxyethyl)phosphine (TCEP) and thiols are not tolerated.
− Inactivation of residual SIA sites may be necessary in some cases.

**Protocol II: Excess of peptide**
+ A limited fraction of cysteine dimers does not reduce the conjugation ratio.
+ Conjugates with different peptides can be prepared with equal, controlled conjugation ratios.
+/− SIA conjugates of a very precise activation level are difficult to prepare. However, since most carrier proteins and SIA are relatively cheap reagents, these exploratory experiments (different ratios) do not consume any of the expensive peptide.
+/− TCEP and thiols are not tolerated. However, in most cases, reductants are not needed.
− Higher amounts of peptide are necessary.

Until now, whether these protocols can be applied to other proteins and peptides had not been evaluated. The activation step is based on well-established NHS chemistry. Hence, it can be assumed that SIA can be used in all cases, in which NHS conjugations had been successful before. There are some indications that good mixing is important for homogeneous derivatization yields. Due to very different numbers of accessible amino groups in proteins, some general adaptations might be necessary. The maximum number of accessible lysine side chains is of particular relevance. The peptide conjugation step can be expected to be highly predictable, as long as the thiol-containing peptide can be synthesized, purified and is sufficiently soluble in an adequate reaction buffer.

In most cases, our approach leads to products with a distribution of different peptide densities and conjugation sites, if the accessible amino groups on the protein cannot be controlled otherwise.

We suggest that the commercially available linker SIA should be considered for most peptide–protein conjugations. Today, only very few protocols are known, which directly lead to bioconjugates of predefined average stoichiometry. SIA shows many advantages in relation to the traditional maleimide linkers.

## Figures and Tables

**Table 1:** (**a**) Half-life of Succinimidyl Iodoacetate (SIA) at different pH values; (**b**) Half-life of *N*-(β-Maleimidopropyloxy) succinimide ester (BMPS) at different pH values.

pH	t_1/2_ [s]
**(a)**	
5	180
6	110
7	36
**(b)**	
8	550
9	150

**Table 2:** Influence of repeated SIA additions to BSA on activation level.

Activation Step	*m*/*z*	Theoretical Ratio	Experimental Ratio
		(Sequential Addition)	(Cumulative)
0	66,441	0	0
1	69,417	60	17.7
2	70,942	60	26.8
3	71,640	60	30.9
4	72,095	60	33.7
5	72,316	60	35.0

**Table 3:** Influence of SIA excess on activation level of bovine serum albumin BSA at pH 7.4.

*m/z*	Theoretical Ratio	Experimental Ratio
66,395	0	0
67,753	20	8.1
68,621	40	13.3
69,585	60	19.0

**Table 4:** Protocol I: Quantitative conjugation ratios obtained by different peptide ratios.

BSA	BSA + SIA	Activ. Level	BSA + SIA + Peptide	calc. Ratio	exp. Ratio
(*m*/*z*)	(*m/z*)	SIA/BSA	(*m*/*z*)	Peptides/BSA	Peptides/BSA
66,448	72,945	38.7	80,561	10	10.8
66,448	72,945	38.7	84,159	15	16.0
66,448	72,945	38.7	94,608	30	30.8
